# Early acute kidney injury and sepsis: a multicentre evaluation

**DOI:** 10.1186/cc6863

**Published:** 2008-04-10

**Authors:** Sean M Bagshaw, Carol George, Rinaldo Bellomo, the ANZICS Database Management Committee

**Affiliations:** 1 Division of Critical Care Medicine, Faculty of Medicine and Dentistry, 3C1.12 Walter C Mackenzie Centrel, University of Alberta Hospital, University of Alberta, 8440-112 St NW, Edmonton, Alberta, T6G2B7 Canada; 2 Department of Intensive Care, Austin Hospital, Melbourne, Australia; 3 Australia New Zealand Intensive Care Society (ANZICS) Adult Patient Database (APD), 10 Ievers St, Melbourne, Australia 3052; 4 Department of Medicine, Melbourne University, Grattan St, Melbourne, Australia 3052

## Abstract

**Introduction:**

We conducted a study to evaluate the incidence, risk factors and outcomes associated with early acute kidney injury (AKI) in sepsis.

**Methods:**

The study was a retrospective interrogation of prospectively collected data from the Australian New Zealand Intensive Care Society Adult Patient Database. Data were collected from 57 intensive care units (ICUs) across Australia. In total, 120,123 patients admitted to ICU for more than 24 hours from 1 January 2000 to 31 December 2005 were included in the analysis. The main outcome measures were clinical and laboratory data and outcomes.

**Results:**

Of 120,123 patients admitted, 33,375 had a sepsis-related diagnosis (27.8%). Among septic patients, 14,039 (42.1%) had concomitant AKI (septic AKI). Sepsis accounted for 32.4% of all patients with AKI. For septic AKI stratified by RIFLE (risk of renal failure, injury to the kidney, failure of kidney function, loss of kidney function and end-stage kidney disease) category, 38.5% of patients belonged to the risk category, 38.8% to the injury category and 22.7% to the failure category. Septic AKI patients had greater acuity of illness (*P *< 0.0001), lower blood pressure (*P *< 0.0001), higher heart rates (*P *< 0.0001), worse pulmonary function measures by arterial oxygen tension/fraction of inspired oxygen ratio (*P *< 0.0001), greater acidaemia (*P *< 0.0001) and higher white cell counts (*P *< 0.0001) compared with patients with nonseptic AKI. Septic AKI was also associated with greater severity of AKI (RIFLE category injury or failure) compared with nonseptic AKI. Septic AKI was associated with a significantly higher crude and co-variate adjusted mortality in the ICU (19.8% versus 13.4%; odds ratio 1.60, 95% confidence interval 1.5 to 1.7; *P *< 0.001) and in hospital (29.7% versus 21.6%; odds ratio 1.53, 95% confidence interval 1.46 to 1.60; *P *< 0.001) compared with nonseptic AKI. Septic AKI was associated with higher ICU and hospital mortality across all strata of RIFLE categories. Septic AKI patients had longer durations of stay in both ICU and hospital across all strata of RIFLE categories.

**Conclusion:**

Septic AKI is common during the first 24 hours after ICU admission. Patients with septic AKI are generally sicker, with a higher burden of illness, and have greater abnormalities in acute physiology compared with patients with nonseptic AKI. Moreover, septic AKI is independently associated with higher odds of death and longer duration of hospitalization.

## Introduction

Acute kidney injury (AKI) is a common clinical problem in intensive care unit (ICU) patients and independently predicts poor outcome [1-4]. Recently, two large multicentre cohort studies [5,6] reported the occurrence of AKI in an estimated 36% of all patients admitted to the ICU. Moreover, additional observational data indicate that the incidence of AKI is rising [7,8].

This large and increasing burden of AKI has in part been attributed to shifts in patient demographics (older, more co-morbid illness), severity of illness (multiple organ dysfunction syndrome) and AKI associated with complex interventions (organ transplantation) [9,10]. Consequently, the aetiology of AKI in critically ill patients is often multifactorial. However, sepsis has consistently been found to be a leading contributing factor to AKI in critical illness [11-18]. Discriminating between AKI of septic and nonseptic origin may have clinical relevance [19]. Evolving data suggest that septic AKI may be characterized by a distinct pathophysiology [9,20-22]. For that reason, septic AKI may be associated with important differences in terms of patient characteristics, response to interventions and clinical outcomes when compared with nonseptic precipitants of AKI.

Regrettably, to date, relatively few studies have focused on describing the epidemiology of septic AKI in critically ill patients [12-18]. Two multicentre studies [12,15] have shown that 46% to 48% of all AKI in critically ill patients can be attributed to sepsis [12,15]. Alternatively, those studies primary focused on sepsis have described that an estimated 10% to 50% of patients develop AKI [13,14,16-18].

Accordingly, in view of the relatively limited data available on septic AKI and its likely importance, we interrogated the Australian and New Zealand Intensive Care Society (ANZICS) Adult Patient Database (APD) to obtain information on all critically ill patients with both sepsis and AKI from 57 Australian ICUs over a 5-year period. Our objectives were as follows: to describe and compare the incidence and clinical characteristics of critically ill patients admitted with sepsis, nonseptic AKI and septic AKI; to describe and compare the severity of AKI stratified by sepsis; and to describe and compare the clinical outcomes of sepsis, nonseptic AKI and septic AKI.

## Materials and methods

This was a retrospective analysis of prospectively collected data. We interrogated the ANZICS APD for all adult (age ≥18 years) intensive care unit (ICU) admissions for a duration of 24 hours or longer associated with a primary diagnosis of sepsis from 1 January 2000 to 31 December 2005. In the event of multiple admissions, only the initial ICU admission was considered in order to avoid bias. For those patients who were readmitted within 72-hours after initial discharge, the readmission was considered part of the initial index admission. The ANZICS APD is a high quality clinical and research database that routinely captures clinical, physiological and laboratory data for all patients admitted to the ICU. Physiological and laboratory data are only captured for the index 24 hours after ICU admission. The database also captures both ICU and hospital clinical outcome data. The ANZICS APD contains data from more than 600,000 individual adult admissions to 135 ICUs from 1987 to the present [23]. We selected only those ICUs that had continuously contributed data to the APD during this 5-year period. This cohort included 57 ICUs (19 tertiary referral, 15 metropolitan, 12 regional/rural and 11 private hospitals).

### Operational definitions/identification of cases

The presence of AKI was assessed for within the first 24 hours after admission (early AKI). AKI was classified according to the RIFLE (risk of renal failure, injury to the kidney, failure of kidney function, loss of kidney function and end-stage kidney disease) criteria with a modification of the urine output criteria [24]. For this study, the outcome RIFLE categories loss and end-stage kidney disease were not evaluated. Although urine output was described in 92.5% of all patients, only the cumulative 24-hour output was available and no patient weights were described [5]. Thus, we used a minor modification of the RIFLE urine output criteria as previously described, assuming an average patient weight of 70 kg and dividing patients into the following categories: <35 ml/hour (risk), <21 ml/hour (injury) or <4 ml/hour (failure). Baseline serum creatinine values were estimated using the Modification of Diet in Renal Disease (MDRD) equation, as recommended by the Acute Dialysis Quality Initiative (ADQI) Working Group (assuming a lower limit of normal baseline glomerular filtration rate of 75 ml/minute) and similar to previous studies [5,6]. For analysis, patients were assigned to their worst RIFLE category according to either serum creatinine or urine output criteria. As data only for the first 24 hours after admission are available, we use the term 'early' to describe the septic AKI syndrome reported in this study. Sepsis was defined using consensus criteria [25,26].

### Data collection

Standard demographic, clinical and physiological data were retrieved. Demographic information included age, sex, and dates and source of admission. Clinical data encompassed primary diagnosis, surgical status, presence of co-morbidities and need for mechanical ventilation. Physiological data included Glasgow Coma Scale score, vital signs, arterial oxygen tension (PaO_2_)/fraction of inspired oxygen (FiO_2_) ratio, serum pH, serum sodium, potassium, bilirubin, haematocrit and white cell count. Data on kidney function included serum creatinine, urea and urine output [23]. Severity of illness was assessed using the Acute Physiology and Chronic Health Evaluation (APACHE) II, APACHE III and Simplified Acute Physiology Score (SAPS) II systems [27,28]. Pre-existing co-morbidities were defined by use of the chronic health evaluation for APACHE II, APACHE III and SAPS II systems, as outlined in the ANZICS APD data dictionary [23].

### Statistical analysis

Analysis was performed using Intercooled Stata (Stata Corp, College Station, TX, USA). In the event of missing data values, data were not replaced. Normally or near normally distributed variables are reported as means with standard deviations and compared using Student's *t*-test, analysis of variance, or simple linear regression. Non-normally distributed continuous data are reported as medians with interquartile ranges (IQRs) and compared using Mann-Whitney U-test or Kruskal-Wallis test. Categorical data were reported as proportions and compared using Fisher's exact test. Multivariable logistic regression analysis was used to assess the association of septic AKI with ICU and hospital mortality. *A priori *selected variables included age, sex, co-morbidity, sepsis diagnosis, AKI diagnosis, need for mechanical ventilation, APACHE II score and hospital site. Model fit was assessed by the goodness-of-fit test and discrimination was assessed using the area under the receiver operator characteristic curve. Data are presented as odds ratios (ORs) with 95% confidence intervals (CIs). *P *< 0.05 was considered statistically significant for all comparisons.

## Results

In total, 120,123 patients were admitted to the 57 ICUs during the 5-year study. These patients had a mean ± standard deviation age of 61.7 ± 17.5 years, 59% were male, 28.6% had co-morbid disease, and the mean ± standard deviation APACHE II scores were 16.9 ± 7.7.

### Sepsis

Sepsis was the primary admission diagnosis in 33,375 patients (estimated cumulative incidence of 27.8%). Septic patients were generally younger (*P *< 0.0001), were more likely to be female (*P *< 0.0001), with a greater burden of co-morbid disease (*P *< 0.0001) and higher severity of illness scores (*P *< 0.0001), and were more likely to have been admitted to the ICU for a medical indication (*P *< 0.0001), as compared with nonseptic patients (Tables 1 and 2). Of those patients with sepsis, 42.1% had concomitant AKI.

**Table 1:** Patient demographics at ICU admission stratified by admission diagnosis of sepsis and AKI

Characteristics	None (n = 57,392)	Sepsis, no AKI (n = 19,336)	Nonseptic AKI (n = 29,356)	Septic AKI (n = 14,039)	*P*
Age (years)	58.6 (17.8)	57.4 (17.9)	68.1 (15.2)	66.7 (15.5)	<0.0001
Male sex (%)	61.9	59.3	57.1	54.6	<0.0001
Co-morbid disease (%)	26.4	27.5	31.1	34	<0.0001
Cardiovascular	15.4	11.8	18.4	15.1	<0.0001
Respiratory	7.7	8.0	9.3	9.8	<0.0001
Liver	1.8	2.2	2.6	3.5	<0.0001
Immunocompromised	3.5	6.6	4.4	9.0	<0.0001
Haematologic malignancy	1.0	2.5	1.5	3.9	<0.0001
Metastatic cancer	2.8	3.7	2.4	3.5	0.57
Surgical (%)	62.1	37.9	49.0	16.3	<0.0001
Emergency	27.3	33.8	36.9	49.9	<0.0001

AKI, acute kidney injury; ICU, intensive care unit.

**Table 2:** Summary of acute physiology and laboratory parameters by sepsis and AKI

	None (n = 57,392)	Sepsis, no AKI (n = 19,336)	Nonseptic AKI (n = 29,356)	Septic AKI (n = 14,039)	*P*
APACHE II score (mean [SD])	14.4 (6.4)	15.4 (7.0)	20.0 (7.9)	22.3 (8.1)	<0.0001
GCS score (median [IQR])	15 (14–15)	15 (13–15)	15 (13–15)	15 (12–15)	0.001
Mean arterial pressure (mmHg; mean [SD])	84.3 (26.9)	86.5 (27.7)	81.3 (28.2)	77.5 (28.3)	<0.0001
Heart rate (beats/minute; mean [SD])	91 (31)	96 (32)	96 (33)	104 (35)	<0.0001
Respiratory rate (breaths/minute; mean [SD])	18 (10)	20 (11)	20 (10)	23 (11)	<0.0001
PaO_2_/FiO_2 _ratio (mean [SD])	287 (154)	262 (145)	260 (153)	230 (140)	<0.0001
PaCO_2 _(mmHg; mean [SD])	42.7 (12.4)	42.5 (13.1)	43.2 (14.5)	42.0 (14.5)	<0.0001
Temperature (°C; mean [SD])	36.7 (1.5)	36.8 (1.6)	36.6 (1.6)	36.9 (1.7)	<0.0001
Mechanical ventilation (%)	53.3	49.5	52.6	49.2	<0.0001
pH (mean [SD])	7.35 (0.1)	7.35 (0.1)	7.30 (0.1)	7.29 (0.1)	<0.0001
Bilirubin (mmol/l; median [IQR])	12 (6–18)	12 (6–18)	13 (6–19)	14 (7–21)	0.0001
Albumin (g/l; mean [SD])	28.2 (7.4)	27.8 (7.6)	26.2 (7.6)	25.1 (7.4)	<0.0001
Haematocrit (%; median [IQR])	0.33 (0.28–0.39)	0.34 (0.28–0.39)	0.31 (0.27–0.39)	0.32 (0.27–0.38)	0.0001
White cell count (× 10^9 ^cells/ml; mean [SD])	13.4 (9.7)	14.2 (13.8)	15.0 (11.5)	16.8 (14.7)	<0.0001
Potassium (mmol/l; mean [SD])	4.2 (0.7)	4.1 (0.7)	4.5 (1.0)	4.4 (1.0)	<0.0001
Creatinine (μmol/l; median [IQR])	76 (60–90)	73 (57–90)	141 (113–200)	165 (124–254)	0.0001
Urea (mmol/l; median [IQR])	5.3 (4–7)	5.6 (4–7.8)	10.4 (7.3–16.1)	13.2 (8.9–19.8)	0.0001
Urine output (l/24 hours; median [IQR])	2.12 (1.54–2.97)	2.05 (1.50–2.98)	1.40 (0.68–2.28)	1.39 (0.66–2.30)	0.0001

APACHE, Acute Physiology and Chronic Health Evaluation; FiO_2_, fraction of inspired oxygen; GCS, Glasgow Coma Scale; IQR, interquartile range; PaCO_2_, arterial oxygen tension; PaO_2_, arterial oxygen tension; SD, standard deviation.

### Early acute kidney injury

Early AKI was present in 43,395 patients (estimated cumulative incidence 36.1%). Stratified by the RIFLE criteria, 16.3% had risk, 13.6% had injury and 6.3% had failure. Compared with non-AKI patients, those with AKI were older (*P *< 0.0001), were more likely to be female (*P *< 0.0001), had more co-morbid disease (*P *< 0.0001), had higher severity of illness scores (*P *< 0.0001) and were more likely to be admitted to the ICU for medical disease (Tables 1 and 2). Of those patients with AKI, 32.4% had a primary diagnosis of sepsis.

### Septic acute kidney injury

Septic AKI was present in 14,039 patients at ICU admission (estimated cumulative incidence 11.7%). For septic AKI stratified by RIFLE criteria, 38.5% had risk, 38.8% had injury and 22.7% had failure. The clinical characteristics, acute physiology and laboratory parameters for septic AKI compared with sepsis only and nonseptic AKI are shown in Tables 1 and 2. Patients with septic AKI had greater acuity of illness (*P *< 0.0001), lower blood pressure (*P *< 0.0001), higher heart rates (*P *< 0.0001), worse pulmonary function measures by PaO_2_/FiO_2 _ratio (*P *< 0.0001), greater acidaemia (*P *< 0.0001) and higher white cell counts (*P *< 0.0001) when compared with either sepsis with no AKI and nonseptic AKI. Septic AKI was also associated with greater severity of AKI (RIFLE category injury or failure) compared with nonseptic AKI (Table 3).

**Table 3:** Incidence of AKI stratified by RIFLE criteria and by an admission diagnosis of sepsis

RIFLE category^a^	Nonseptic (n = 86,748)	Septic (n = 33,375)
None (%)	66.2	57.9
Risk (%)	16.3	16.2
Injury (%)	12.6	16.3
Failure (%)	5.0	9.6
Any RIFLE category (%)	33.8	42.1

^a^Classification into RIFLE category based on fulfilling worst criteria for either serum creatinine or urine output. AKI, acute kidney injury; RIFLE, risk of renal failure, injury to the kidney, failure of kidney function, loss of kidney function and end-stage kidney disease

### Mortality

**Figure 1:**
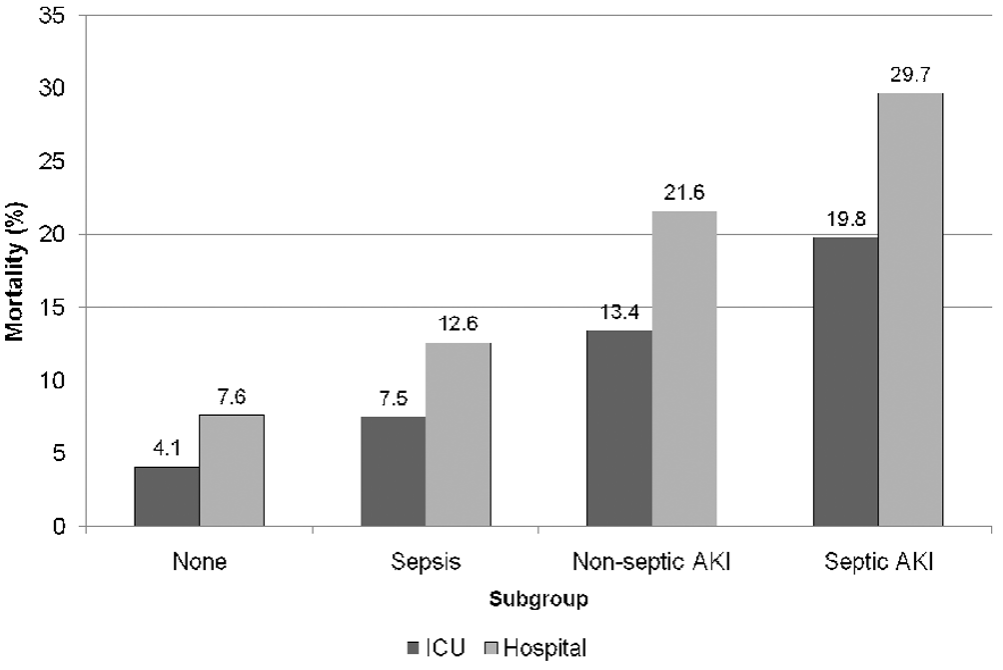
Crude ICU and hospital mortality stratified by subgroups. The subgroups of patients were control, sepsis, acute kidney injury (AKI) and septic AKI. ICU, intensive care unit.

**Figure 2:**
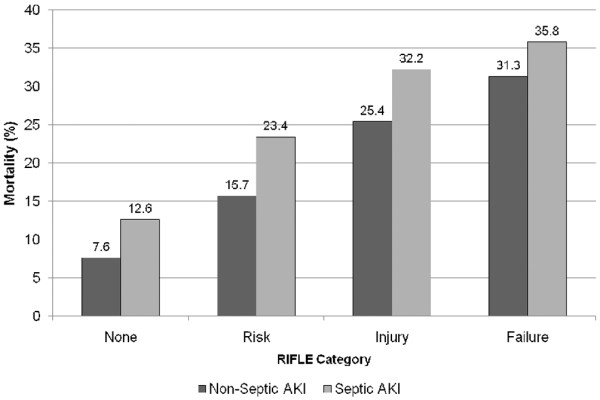
Crude hospital mortality for septic and non-septic AKI stratified by RIFLE category. For each comparison of nonseptic versus septic acute kidney injury (AKI), *P *< 0.0001. RIFLE, risk of renal failure, injury to the kidney, failure of kidney function, loss of kidney function and end-stage kidney disease.

Septic AKI was associated with a significantly higher crude ICU (19.8% versus 13.4%; OR 1.60, 95% CI 1.5 to 1.7; *P *< 0.001) and in-hospital (29.7% versus 21.6%; OR 1.53, 95% CI 1.46 to 1.60; *P *< 0.001) mortality when compared with nonseptic AKI. Similarly, septic AKI, when compared with sepsis only, was associated with significantly higher mortality in the ICU (19.8% versus 7.5%; OR 3.07, 95% CI 2.9 to 3.3; *P *< 0.001) and in hospital (29.7% versus 12.6%; OR 2.93, 95% CI 2.8 to 3.1; *P *< 0.001; Figure 1 and Table 4). Septic AKI, compared with nonseptic AKI, was consistently associated with higher ICU and hospital mortality across all strata of RIFLE categories (Figure 2). In multivariable analysis, after adjustment for co-variates, sepsis, nonseptic AKI and septic AKI were all found to be significantly associated with higher ICU and hospital mortality (Table 4).

**Table 4:** Summary of crude and co-variate adjusted odds ratios for mortality for septic AKI admitted to ICU

Category	ICU mortality		Hospital mortality	
	
	Crude OR (95% CI)	Adjusted OR (95% CI)^a^	Crude OR (95% CI)	Adjusted OR (95% CI)^b^
None	1.0	1.0	1.0	1.0
Sepsis	1.89 (1.77–2.03)	1.47 (1.36–1.60)	1.75 (1.65–1.84)	1.40 (1.31–1.49)
Nonseptic AKI	3.64 (3.45–3.84)	1.53 (1.44–1.63)	3.36 (3.22–3.50)	1.43 (1.36–1.50)
Septic AKI	5.81 (5.48–6.17)	1.69 (1.57–1.82)	5.12 (4.89–5.38)	1.54 (1.45–1.64)

^a^Area under the receiver operator characteristic (AuROC) 0.86, goodness-of-fit *P *= 1.0. ^b^AuROC 0.84, goodness-of-fit *P *= 1.0. AKI, acute kidney injury; CI, confidence interval; ICU, intensive care unit; OR, odds ratio; RIFLE, risk of renal failure, injury to the kidney, failure of kidney function, loss of kidney function and end-stage kidney disease

### Secondary outcomes

**Figure 3:**
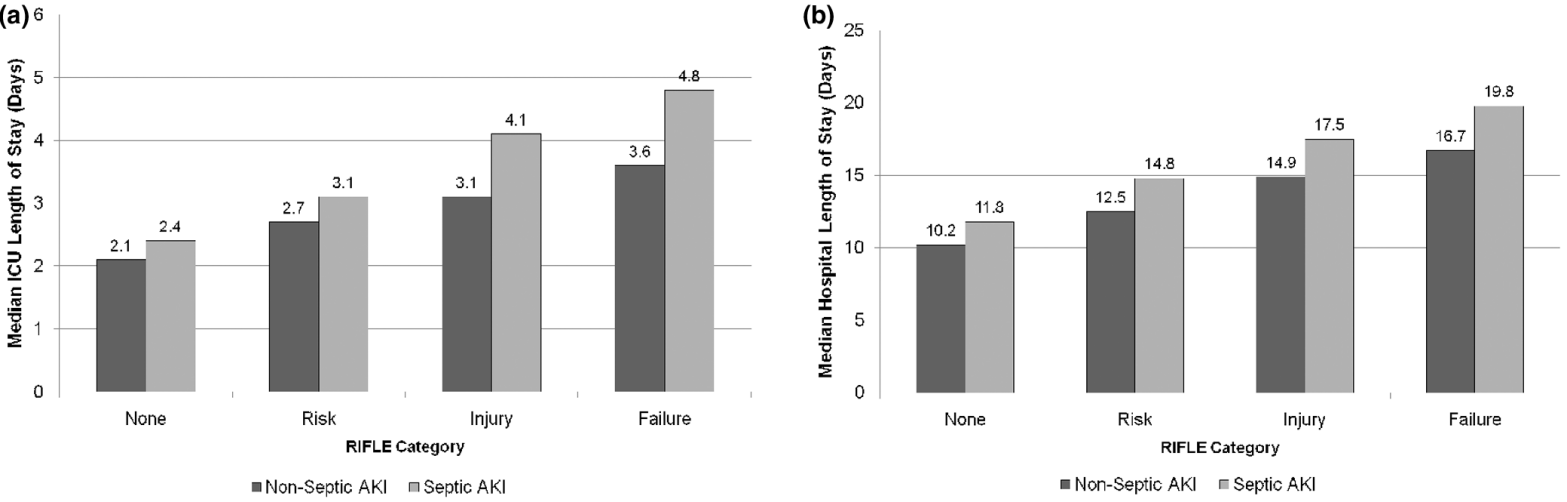
ICU and hospital length of stay. **(a) **Intensive care unit (ICU) length of stay for acute kidney injury (AKI) patients stratified by sepsis and RIFLE category. For each comparison of nonseptic versus septic AKI, *P *< 0.0001. **(b) **Hospital length of stay for AKI patients stratified by sepsis and RIFLE category. For each comparison of nonseptic versus septic AKI, *P *< 0.0001. RIFLE, risk of renal failure, injury to the kidney, failure of kidney function, loss of kidney function and end-stage kidney disease.

In survivors to ICU and hospital discharge, sepsis, nonseptic AKI and septic AKI were all associated with progressively longer durations of stay in both ICU and hospital (Table 5). Similarly, septic AKI, as compared with nonseptic AKI, was consistently associated with longer durations of stay in both ICU and hospital across all strata of RIFLE categories (Figures 3a and 3b). In addition, those with septic AKI were found to be less likely to be discharged home and more likely to be transferred to another acute care hospital or long-term rehabilitation centre when compared with patients with nonseptic AKI, sepsis only, or neither (Table 5).

**Table 5:** Secondary outcomes in ICU patients stratified by sepsis and AKI

Clinical outcome	None (n = 57,392)	Sepsis, no AKI (n = 19,336)	Nonseptic AKI (n = 29,356)	Septic AKI (n = 14,039)	*P*
ICU length of stay (days)					
Dead (median [IQR])	3.9 (2.1–8.1)	4.6 (2.3–9.5)	3.7 (1.9–7.7)	3.9 (2.0–8.8)	0.0001
Alive (median [IQR])	2.1 (1.6–3.8)	2.4 (1.6–4.8)	2.8 (1.8–5.2)	3.8 (2.0–7.6)	0.0001

Hospital length of stay (days)					
Dead (median [IQR])	10.2 (4.4–23.0)	10.6 (4.8–23.0)	9.0 (3.7–20.2)	9.5 (3.7–20.9)	0.0001
Alive (median [IQR])	10.2 (6.9–18.2)	11.8 (6.7–22.7)	13.9 (8.1–25.1)	16.7 (9.5–30.9)	0.0001

Discharge location of survivors (%)					
Home	86.7	81	79.9	75.6	<0.0001
Transfer to other hospital	4.5	5.9	7.3	8.5	<0.0001
Rehabilitation/long-term care	8.8	13.1	12.8	15.9	<0.0001

AKI, acute kidney injury; ICU, intensive care unit; IQR, interquartile range.

## Discussion

We conducted a large multicentre epidemiological ICU study of more than 14,000 cases of sepsis complicated by early AKI. Our principal objectives were to describe the incidence, clinical characteristics and outcomes associated with septic AKI and to compare early septic AKI patients with critically ill patients with nonseptic AKI, sepsis only and neither sepsis nor AKI.

First, we found that early septic AKI is common and present in nearly 12% of all ICU admissions. Importantly, of those admitted with a primary diagnosis of sepsis, 42% had concomitant AKI. Similarly, for those with AKI, an estimated 32% had sepsis as a contributing factor. Second, our findings suggest that septic AKI patients are clinically distinct and have features that differentiate them from patients with nonseptic AKI.

Septic AKI patients are older and have more co-morbid disease. Septic AKI was more likely to be associated with medical admissions; however, if the admission was surgical, then septic AKI was more likely to be associated with an emergency surgical admission.

Septic AKI was also characterized by greater acuity of illness as demonstrated by severity of illness scores and greater aberrancy in vital signs, markers of inflammation and blood chemistry. Third, these distinguishing features of septic AKI appeared to translate into relevant differences in clinical outcomes when compared with nonseptic AKI. For example, septic AKI was associated with greater risks for both ICU and hospital death. This was consistent across all strata of AKI severity when stratified by RIFLE category. Likewise, septic AKI contributed to significantly longer durations of stay in both ICU and hospital.

These findings are largely consistent with and extend data from prior investigations into septic AKI. Observational studies have shown that the incidences of sepsis and AKI are increasing [7,8,10,29,30]. Small single centre studies have found that 11% to 37% of all septic patients have concomitant AKI [13,14,18]. The multicentre European Sepsis Occurrence in Acutely Ill Patients (SOAP) study found that 51% of septic patients developed AKI, defined by a Sequential Organ Failure Assessment score above 2 (serum creatinine >177 μmol/l) [17]. More recently, in a 1-day point prevalence survey for severe sepsis/septic shock from 454 ICUs in Germany, Oppert and coworkers [16] reported concomitant AKI in 41.4% of septic patients. Likewise, two large multicentre observational studies of critically ill patients with AKI [12,15] found sepsis to be a contributing factor in 46% to 48% of episodes of AKI. However, these studies are somewhat limited by their inclusion of only septic patients or only AKI patients, and therefore they provide a potentially biased comparison.

Our data are largely comparable, but we can further illustrate the overall high burden of septic AKI in relation to all ICU admissions (11.7% overall). Moreover, we can show both the high incidence of early AKI accompanying sepsis (42.1%) and sepsis contributing to early AKI (32.4%). In addition, our study is the first to date to compare clinical characteristics and outcomes between septic AKI and nonseptic AKI, sepsis only, and a control cohort with neither sepsis nor AKI. We believe that the generalizability of our study is further strengthened by incorporating data from multiple centres and across a range of hospital types. A reasonable inference from these accumulated data is that sepsis has clearly surfaced as the most significant predictor of AKI in critically ill patients and that the occurrence of septic AKI is likely to increase further.

The findings of our study further support the concept that discriminating septic and nonseptic AKI may have clinical importance. In other words, septic AKI may differ from AKI induced by other factors and from sepsis not complicated by AKI [19]. The mechanisms that account for these differences remain speculative, but they may relate to the physiological and immune consequences of either sepsis or AKI alone or of their additive and complex interplay. Studies have generally found septic AKI to be associated with older age, greater co-morbid disease, nonsurgical disease, greater severity of illness, more aberrancy in haemodynamic parameters, greater need for mechanical ventilation and vasopressor support, and greater disturbances in inflammation, haematology, and acid-base homeostasis [12,14-16,18]. Two studies [12,13] have also found septic AKI to be associated with higher central venous filling pressures and lower urine output when compared with nonseptic AKI. Moreover, the concept that septic AKI may have a distinct pathophysiology is supported not only by experimental data and evidence from small clinical studies [9,20,31-34] but also by epidemiological data showing 'dose-response' trends in incidence rates and outcomes for septic AKI by either severity of sepsis or AKI.

Rangel-Frausto and coworkers [11] were the first to show how the incidence of AKI, defined by need for renal replacement therapy, increased significantly according to whether patients had sepsis (24%), severe sepsis (39%), or septic shock (89%). Lopes and colleagues [14] showed that the rate of AKI increased from 29% to 51% when categorized from severe sepsis to septic shock. This study also found that the relative severity of AKI in septic shock was worse, with a greater proportion classified RIFLE category failure when compared with those with severe sepsis (64% versus 36%). We contend that the differences in pathophysiology between AKI due to sepsis and other aetiologies (ischaemia, toxin, cardiopulmonary bypass) may have important clinical implications for detection of early kidney injury, changes to kidney function and trajectory of AKI, along with the application of potential interventions to attenuate injury and promote kidney recovery.

Furthermore, all prior studies describing septic AKI have consistently concluded that septic AKI contributes to markedly higher mortality than either nonseptic AKI or sepsis alone [12-16,18]. These studies are in general agreement with our findings. Moreover, we were also able to show a 'dose-response' increase in mortality with greater severity of AKI by RIFLE category in sepsis compared with nonsepsis (Figure 2). We contend this also provides further support for the clinical relevance and robustness of the RIFLE classification system. Similarly, Neveu and coworkers [15] previously found a near linear increase in mortality for AKI when categorized as nonseptic or associated with either sepsis syndrome or septic shock. This 'dose-response' trend for septic AKI was likewise apparent when evaluating durations of stay in ICU and hospital for survivors by severity of AKI. These data, within the context of prior investigations, logically imply that septic AKI may be distinct, may behave differently and may independently portend a worse prognosis. We contend that these findings have relevance for the management of the septic patient with AKI. Accordingly, a diagnosis of septic AKI may deserve consideration for separate stratification of randomization and *a priori *identification for subgroup analysis when applying therapeutic strategies and/or designing future research investigations of the treatment of AKI.

There are limitations to our study. First, we estimated the occurrence of AKI at or within the first 24 hours of ICU admission only. Thus, we are unable to comment on the occurrence and outcomes for patients with ICU-acquired AKI or sepsis, which may be associated with a worse outcome [35]. We recognize this may result in an underestimate of the true cumulative incidence of septic AKI. However, we believe that our data provide an important approximation of the disease burden associated with septic AKI. Second, we were unable to determine baseline creatinine values or the prevalence of chronic kidney disease with the exception of those with end-stage renal disease. However, this problem is not uncommon. We thus calculated an estimate of baseline function by use of the MDRD equation as recommended by the ADQI group [24]. Nonetheless, these factors may contribute to a misclassification of some patients and result in an overestimate of the occurrence of septic AKI. Third, we were unable to describe changes over time in kidney function or transition between RIFLE criteria. Finally, we were unable to describe the association of initial RIFLE category to other clinical outcomes such as the proportion of patients receiving renal replacement therapy, long-term survival, or renal recovery beyond hospital discharge. Thus, whether septic AKI contributes to downstream morbidity and mortality conditional on hospital survival remains likely but unknown.

In summary, we conducted a multicentre observational study of the incidence and outcomes of septic AKI in a large heterogeneous cohort of critically ill patients. We showed that septic AKI is common and is associated with a higher burden of illness and greater abnormalities in acute physiology and laboratory parameters compared with either nonseptic AKI or sepsis alone. We have further shown that septic AKI confers an important and independent increase in risk for hospital death that exceeds that of either nonseptic AKI or sepsis alone. In survivors, septic AKI was also associated with prolonged ICU and hospital stays, and a greater likelihood of discharge to rehabilitation or another acute care facility. We contend that these data support the emerging concept that septic AKI may represent a unique pathophysiological condition. Moreover, we believe that future investigations should consider these differences in AKI pathophysiology when applying potential therapeutic interventions.

## Key messages

• Early septic AKI is very common.

• Early septic AKI is characterized by significant differences in acute physiology and severity of illness when compared with nonseptic AKI or sepsis alone.

• Early septic AKI contributes to higher mortality and greater durations of stay in ICU and hospital for survivors.

• Septic AKI may represent a unique pathophysiologic condition and, as such, may deserve consideration for separate stratification of randomization and *a priori *identification for subgroup analysis when applying therapeutic strategies and/or designing future research investigations.

## Abbreviations

ADQI = Acute Dialysis Quality Initiative; AKI = acute kidney injury; ANZICS = Australian and New Zealand Intensive Care Society; APACHE = Acute Physiology and Chronic Health Evaluation; APD = Adult Patient Database; CI = confidence interval; FiO_2 _= fraction of inspired oxygen; ICU = intensive care unit; MDRD = Modification of Diet in Renal Disease; OR = odds ratio; PaO_2 _= arterial oxygen tension; RIFLE = risk of renal failure, injury to the kidney, failure of kidney function, loss of kidney function and end-stage kidney disease; SAPS = Simplified Acute Physiology Score.

## Competing interests

The authors declare that they have have no competing interests.

## Authors' contributions

SMB and RB were responsible for study conception and design. CG was responible for acquisition of data. SMB and RB analyzed and interpreted the data. SMB drafted the manuscript. SMB, CG and RB critically revized the manuscript.
